# Effectiveness of quadrivalent human papillomavirus vaccination against high-grade cervical lesions by age and doses: a population-based cohort study

**DOI:** 10.1016/j.lanepe.2024.101178

**Published:** 2025-01-05

**Authors:** Shiqiang Wu, Alexander Ploner, Ana Martina Astorga Alsina, Yunyang Deng, Lina Ask Schollin, Jiayao Lei

**Affiliations:** a Department of Medical Epidemiology and Biostatistics, Karolinska Institutet, Stockholm, Sweden; b Unit for Vaccination Programmes, Department of Public Health Analysis and Data Management, Public Health Agency of Sweden, Solna, Sweden; c Department of Women's and Children's Health, Karolinska Institutet, Stockholm, Sweden; d Department of Clinical Science, Intervention and Technology, Karolinska Institutet, Stockholm, Sweden

**Keywords:** HPV vaccine, Cervical cancer, Cervical lesions, Vaccine effectiveness, Public health

## Abstract

**Background:**

One or two-dose schedule for human papillomavirus (HPV) vaccination has been recommended by the World Health Organization and used in many vaccination programs. We aimed to comprehensively evaluate the effectiveness of quadrivalent HPV vaccine against high-grade cervical lesions by age at vaccination and number of doses received.

**Methods:**

This cohort study included 2,200,495 females aged 10–35 years old who were residents of Sweden between 2006 and 2022, with 584,676 (26.6%) receiving at least one dose of quadrivalent HPV vaccine. We used Poisson regression models to estimate the incidence rate ratios (IRR) comparing the incidence rate of high-grade cervical lesions in relation to age at vaccination and doses.

**Findings:**

In girls initiating vaccination before age 15, we observed IRRs of 0.42 (95% CI 0.33–0.52) after one-dose, 0.54 (0.47–0.63) after two-dose, and 0.50 (0.47–0.53) after three-dose. The IRRs were 0.60 (95% CI 0.52–0.70), 0.55 (0.49–0.62), and 0.54 (0.52–0.56) after one, two or three doses for girls who initiated vaccination age 15–17. For women who initiated vaccination after age 20, higher doses may be needed to achieve a statistically significant risk reduction.

**Interpretation:**

Receiving one or two doses of HPV vaccines prior to age 17, especially for those initiating before age 15, has comparable effectiveness against high-grade cervical lesions with those who received three doses.

**Funding:**

10.13039/501100004359Swedish Research Council, 10.13039/501100006636Swedish Research Council for Health, Working Life and Welfare, and 10.13039/501100004047Karolinska Institutet.

**Research in context**

**Evidence before this study**

We searched PubMed from database inception to 21 August 2024, for all types of articles without language restrictions, using the following search terms: (“hpv vaccin∗” [All Fields] OR “human papillomavirus vaccin∗” [All Fields]) AND (“effectiveness” [All Fields] OR “efficacy” [All Fields]) AND “dose” [All Fields]. Current evidence from two ongoing randomized controlled trials, the Kenya Single-Dose HPV Vaccine Efficacy Trial and the Dose Reduction Immunobridging and Safety Study, indicates that a single dose provides comparable immunogenicity and efficacy against HPV infection to two or three doses in girls aged 9–20 years. Nevertheless, evidence on the efficacy of reduced-dose vaccines against precancerous outcomes from clinical trials is yet to come. Earlier observational studies from Australia, the United States, and Denmark found that women who received a single dose of the HPV vaccine showed comparable effectiveness against precancerous lesions to those who received multiple doses. However, many studies have not demonstrated comparable effectiveness by number of doses, likely due to biases, confounding factors, short follow-up periods, or limited statistical power. Additionally, a recent systematic review and meta-analysis highlighted significant heterogeneity among these observational studies.

**Added value of this study**

We provided dose-specific vaccine effectiveness against high-grade cervical lesions by refined age at vaccination initiation from 17 years of follow-up after the introduction of HPV vaccines. Our findings highlighted that among adolescent females, especially those vaccinated before the age of 15, one or two doses of the vaccine provided comparable protection against high-grade cervical lesions as three doses. For young women who initiated at an older age (above 20 years of age), more doses might be required to achieve significant protection.

**Implications of all the available evidence**

Our findings support the World Health Organization’s recommendation of using a two-dose or one-dose HPV vaccine regimen in girls and young women. As many countries are switching to one-dose regimen to scale up the coverage of HPV vaccination, our findings based on nationwide Swedish registries and real-life programs provided high-quality evidence to support such policy changes and decision-making for HPV vaccination programs globally.

## Introduction

Cervical cancer is the fourth most common cancer among women worldwide in 2022.^1^ As such, the World Health Organization’s (WHO) global strategy aims to eliminate cervical cancer within the next century by scaling up the preventive strategies, including human papillomavirus (HPV) vaccination, screening, and treatment of pre-cancer.^2^ Since the approval of the HPV vaccine in 2006, HPV vaccination has been introduced as part of national immunization services in 143 countries around the world.^3^ Evidence from real-world studies shows the remarkable effectiveness of HPV vaccination in preventing invasive cervical cancer.^4^, ^5^, ^6^ At initial approval, all HPV vaccines were administered through a three-dose regimen.^7^ However, the WHO recommendation later changed to follow a one- or two-dose (with at least 6 months apart) schedule to facilitate global vaccine access and to eliminate cervical cancer.^2^^,^^7^ To date, 53 countries have switched to a single-dose program, while the rest remain using a two-dose schedule in their programs.^3^

Research regarding the comparable efficacy or effectiveness of reduced-dose HPV vaccine remains active, particularly regarding longer-term disease outcomes. Recently, two randomized controlled trials were conducted in Africa, the Kenya single-dose HPV vaccine efficacy trial and the Dose Reduction Immunobridging and Safety Study trial, along with a further immunobridging analysis of both trials, these have demonstrated comparable immunogenicity and efficacy against HPV infection of one dose compared to two or three doses in girls aged 9–20 years.^8^, ^9^, ^10^ However, evaluating disease endpoint efficacy typically requires a long follow-up and large sample size, which are often impractical in trials. To our knowledge, no randomized trials to date have evaluated the efficacy of reduced-dose vaccines against precancerous stage outcomes or cancer itself. Additionally, trials may lack the power for subgroup analyses, such as lower doses for older women. On the other hand, some observational studies have found a comparable effectiveness of reduced-dose vaccine against precancerous lesions in young women.^11^, ^12^, ^13^ However, many of these studies have limited follow-up periods and lack statistical power to examine the interaction of age and doses. Furthermore, a meta-analysis concluded that there was a high degree of heterogeneity among these studies,^13^ which reduced the reliability of the current evidence on single-dose vaccine effectiveness against pre-cancer. In this population-based study, we aim to comprehensively evaluate the effectiveness by age at vaccination and doses in preventing high-grade cervical lesions 17 years after the introduction of HPV vaccination, using high-quality data from nationwide Swedish registries.

## Methods

### Study population

The study population was covered by various vaccine programs and a population-based organized cervical screening program. The first HPV vaccine was approved for prophylactic use and was available in Sweden in late 2006.^14^ Then, from May 2007, the 3-dose HPV vaccine was subsidized for girls aged 13–17 nationwide.^15^ This continued until 2012, when Sweden introduced the free-of-charge school-based HPV vaccination program that targeted girls aged 10–12, alongside a free-of-charge catch-up HPV vaccination program for girls aged 13–18.^15^ From 2015, the school-based program was administered through a two-dose schedule for girls aged 10–12.^15^ Throughout this time, the quadrivalent HPV vaccine was used almost exclusively until 2019, when the nonavalent vaccine came into use instead.^15^ During our study period, women aged 23–64 years are invited to participate in the population-based, organized cervical cancer screening program with invitations at every 3 year for women aged 23–49 and 5 years for women aged 50–64.^16^ Cytology was initially used as the primary screening method, but since 2015, HPV-based screening has been recommended as the primary test in the program.^16^

**Fig. 1:**
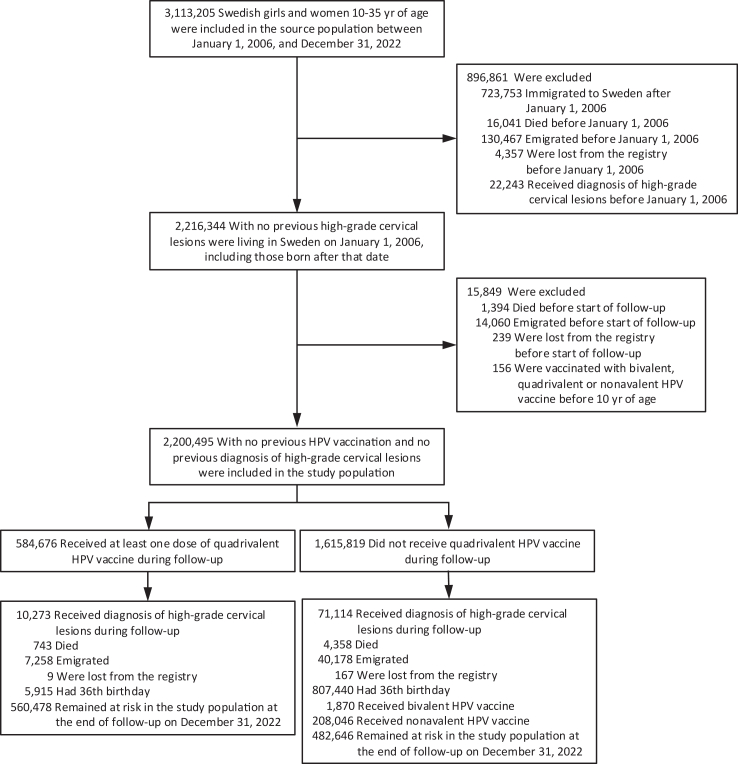
**Study population**.

In this registry-based cohort study, we included females aged 10–35 years old who were residents of Sweden between 2006 and 2022. All enrolled girls and women were followed from January 1, 2006, or their 10th birthdays, whichever came later. We excluded girls or women who had immigrated to Sweden after 1 January 2006, died, emigrated, were lost to follow-up, or had HPV vaccination or been diagnosed with high-grade cervical lesions before entry to the study (Fig. 1). Immigrants who moved to Sweden after 2006 were excluded, because we could not obtain their HPV vaccination records before they immigrated to Sweden. All included girls and women were followed until they were diagnosed with the outcome, died, emigrated from Sweden, were lost to follow-up, received the bivalent or nonavalent HPV vaccination, reached their 36th birthdays or December 31, 2022, whichever came first.

This study was approved by the regional ethical review board in Stockholm (Dnr. 02–556, 2011/921-32, and 2023-06456-02), which determined that written informed consent was not required from the persons included in the study. We followed the Strengthening the Reporting of Observational Studies in Epidemiology (STROBE) reporting guidelines.

### Data collection and linkage

Data used in this study were retrieved from a number of nationwide Swedish population and healthcare registers, and linked at the individual level using the unique Swedish personal identity number (PIN) allocated to all Swedish residents.^17^ The information on the population at risk, immigration, emigration, and death records were retrieved from the Total Population Register (TPR).^18^ Biological or adoptive parents of participants were identified through the Multi-Generation Register.^19^

Data on HPV vaccination was retrieved from the Swedish HPV Vaccination Register (SVEVAC), National Vaccination Register (NVR), and Prescribed Drug Register (PDR). The SVEVAC was a voluntary registry requiring informed consent from the individual or their parent containing HPV vaccine administration dates between 2006 and 2015.^4^ Vaccination doses without consent were registered without PIN in SVEVAC, and can not be linked to other registers. HPV vaccination doses administrated within the school-based vaccination program (up to 18 years of age) have been recorded in NVR since 2013, which is a mandatory report register for all childhood vaccinations in Sweden.^20^^,^^21^ SVEVAC and NVR have served as the main source for HPV vaccine exposure in our study.

The PDR includes drug utilization and expenditures with complete coverage since 2005, and automatically registers Anatomical Therapeutic Chemical codes.^4^^,^^22^ We used vaccine dispensation dates from the PDR to supplement data in the SVEVAC. A vaccine dispensation date was considered as a new HPV vaccination dose if there were at least 14 days apart between a vaccine dispensation and an administered vaccine dose, based on an established algorithm used in previous studies.^4^^,^^23^^,^^24^

### HPV vaccination

Quadrivalent HPV vaccination status was considered a time-varying exposure (Appendix p 2), allowing the same individual to contribute person-time to both the unvaccinated and vaccinated groups. An individual was moved from the unvaccinated group to the vaccinated group upon the administration date of the first quadrivalent HPV vaccine. Vaccinated females could be further stratified according to the number of doses received and the age at first vaccination, respectively. Vaccination dose was assessed as a time-varying exposure as well (Appendix p 2), with levels zero- (unvaccinated), one-, two-, and three-doses, upon the date of administration of each successive dose. Age at vaccination initiation was categorized as 10–14, 15–16, 17–20, and 21–35 according to WHO’s current age cut-offs of recommendations for HPV vaccination regimens,^7^ and the median age of first sexual intercourse for women in Sweden (17 years).^25^

### Outcome definition

We defined the outcome as the first occurrence of a histologically confirmed diagnosis of high-grade cervical lesions. These are defined as cervical intraepithelial neoplasia grades 2 or worse, including adenocarcinoma in situ or worse and invasive cervical cancers. Data were retrieved from the Swedish National Cervical Screening Registry (NKCx), which has virtually complete coverage of cervical screening information and histopathological results in Sweden since 1995, using the corresponding standardized nomenclature of medical diagnoses (SNOMED).^26^

### Covariates

We obtained covariates that might be related to the uptake of HPV vaccination and the underlying risk of high-grade cervical lesions available from nationwide Swedish registers. Attained age and calendar year were included due to their significant relevance to HPV vaccination uptake. Age is also biologically linked to the risk of high-grade cervical lesions, while calendar year captures the changes in screening attendance that could influence the detection of high-grade cervical lesions, as well as potential herd effects. We also obtained county of residence and mother’s country of birth from TPR to capture geographical and cultural variations in health behaviors. Additionally, we retrieved parental education level and annual household income, which can influence vaccine uptake and health behaviors, and also act as partial proxies for lifestyle factors such as smoking status.^27^ This information were retrieved from the Longitudinal Integration Database for Health Insurance and Labor Market Studies (LISA).^28^ Parental education levels were categorized according to educational attainment as low (less than high school), medium (high school), and high (equivalent to university or above). Annual household income levels were categorized into low, medium, and high based on the income level tertiles of the population aged 20 to 65. Given that a significant proportion of individuals were vaccinated during their teenage years, we further included the maternal history of high-grade cervical lesions identified from NKCx to account for parental decisions regarding vaccine uptake, and the underlying risk of cervical disease due to genetic factors or immune-related influences. County of residence, parental education levels, and annual household income levels were measured in the year before study entry.

### Statistical analysis

As descriptive measures, we computed crude incidence rates (IR) using total person-time at risk and the number of high-grade cervical lesion cases diagnosed, stratified by vaccination status, age at vaccination, and number of doses received. We used Poisson regression to model incidence rates adjusted for both attained age and calendar year as time-varying variables, by splitting follow-up time for each individual into shorter intervals, corresponding to one-year changes in age and progression in calendar time simultaneously. We calculated the calendar year by taking the sum of year of birth and the attained age during follow-up. Subsequently, we adjusted for attained age as a natural spline term with 3 degrees of freedom. In the fully adjusted models, we also included calendar year as a categorical variable with one-year levels, county of residence, and parental characteristics, including the maternal history of high-grade cervical lesions, mother’s country of birth, parental highest education level, and annual household income, as described in the previous section. For any covariate with missing values, we introduced an extra category “missing” for modeling.

The adjusted Poisson models were then used to estimate incidence rate ratios (IRR) with a 95% confidence interval (CI) comparing vaccinated and unvaccinated females, as well as different ages at first vaccination and number of doses, respectively. An interaction term between age at vaccination and doses was further included in a separate Poisson model.

To exclude prevalent HPV infections at the time of vaccination, we introduced a buffer period of 12-month intervals between vaccination and case-counting in the main analysis (Appendix p 3). The person-time during this buffer period was included in the previous exposure status.

### Sensitivity analysis

To account for the potential variation in length between prevalent infection when vaccinated and the occurrence of high-grade cervical lesions, we used no buffer period and a buffer period of 6-month, 18-month, and 24-month to compare the vaccine effectiveness by age, doses and its combination. We also performed complete-case analyses based on the 93.5% of our study population who had no missing information (Appendix p 4). Additionally, we repeated the main analysis based on a subset of birth cohorts to account for low vaccination rates as well as low cervical screening participation. We excluded birth cohorts born before 1990 (1970–1989), because the HPV vaccination rates were much lower compared to younger birth cohorts. We excluded the birth cohorts born after 2000 (2000–2012) since they did not reach the age of invitation to cervical screening in Sweden during our study period (23 years old). Subsequently, we performed the analysis by excluding both above two birth cohorts that had low vaccination rates or low screening rates. Finally, the overall vaccine effectiveness was estimated for the various cut-off points of age at first vaccination as an exploratory analysis of how the age is related to the risk of high-grade cervical lesions.

All data management was performed in SAS version 9.4 and data analysis was conducted with Stata version 18 (StataCorp). All statistical tests were two-sided, and we set the level of statistical significance at p < 0.05.

### Role of the funding source

The funders of the study had no role in study design, data collection, data analysis, data interpretation, or writing of the report.

## Results

### Study population and follow-up

Our study included 2,200,495 girls and women aged 10–35 years (Table 1), with a median follow-up time of 10 years (interquartile range [IQR]: 4–16) (Appendix p 5). A total of 584,676 (26.6%) girls and women received at least one dose of quadrivalent HPV vaccine, of which 8.3%, 40.3%, and 51.5% received only one dose, two doses, or three doses, respectively. When stratified by age at vaccination, 69.0% of participants received their first dose before 15 years old. During the study period, there were 71,114 and 10,273 incident cases of high-grade cervical lesions among the unvaccinated and vaccinated groups, respectively (Fig. 1).

**Table 1:** Characteristics of the study population.

Variables	Unvaccinated	Vaccinated^a^	Vaccinated, by dose^b^			Vaccinated, by age at first vaccination			
			One-dose	Two-dose^c^	Three-dose	10–14	15–16	17–20	21–35
Total pop–no. (%)^d^	1,615,819 (73.4)	584,676 (26.6)	48,272 (8.3)	235,396 (40.3)	301,008 (51.5)	403,451 (69.0)	88,706 (15.2)	64,861 (11.1)	27,658 (4.7)
Median years of follow-up (IQR)	8.4 (2.3–15.3)	12.4 (8.7–17.0)	13.5 (11.1–17.0)	8.2 (6.6–9.8)	15.6 (12.4–17.0)	10.2 (7.6–13.0)	17.0 (16.1–17.0)	17.0 (17.0–17.0)	17.0 (16.1–17.0)
Birth cohort–no. (%)^d^									
1970–1979	535,670 (33.2)	708 (0.1)	168 (0.3)	139 (0.1)	401 (0.1)	0 (0.0)	0 (0.0)	0 (0.0)	708 (2.6)
1980–1989	483,012 (29.9)	23,973 (4.1)	4030 (8.3)	4473 (1.9)	15,470 (5.1)	0 (0.0)	4 (0.0)	5961 (9.2)	18,008 (65.1)
1990–1994	171,914 (10.6)	117,836 (20.2)	8218 (17.0)	12,632 (5.4)	96,986 (32.2)	14,353 (3.6)	46,427 (52.3)	48,597 (74.9)	8459 (30.6)
1995–1999	93,049 (5.8)	136,049 (23.3)	14,581 (30.2)	19,570 (8.3)	101,898 (33.9)	85,597 (21.2)	40,371 (45.5)	9609 (14.8)	472 (1.7)
2000–2004	39,413 (2.4)	189,963 (32.5)	16,433 (34.0)	88,106 (37.4)	85,424 (28.4)	187,368 (46.4)	1893 (2.1)	691 (1.1)	11 (0.0)
2005–2012	292,761 (18.1)	116,147 (19.9)	4842 (10.0)	110,476 (46.9)	829 (0.3)	116,133 (28.8)	11 (0.0)	3 (0.0)	0 (0.0)
Mother’s country of birth–no. (%)^d^									
Sweden	1,223,350 (75.7)	494,695 (84.6)	39,346 (81.5)	194,619 (82.7)	260,730 (86.6)	338,743 (84.0)	77,802 (87.7)	55,305 (85.3)	22,845 (82.6)
Other country	264,763 (16.4)	88,010 (15.1)	8672 (18.0)	40,178 (17.1)	39,160 (13.0)	63,680 (15.8)	10,538 (11.9)	9245 (14.3)	4547 (16.4)
Missing	127,706 (7.9)	1971 (0.3)	254 (0.5)	599 (0.3)	1118 (0.4)	1028 (0.3)	366 (0.4)	311 (0.5)	266 (1.0)
Highest parental education level–no. (%)^d^									
Low	127,466 (7.9)	15,715 (2.7)	1869 (3.9)	6414 (2.7)	7432 (2.5)	10,503 (2.6)	2019 (2.3)	2095 (3.2)	1098 (4.0)
Middle	696,471 (43.1)	226,365 (38.7)	20,379 (42.2)	85,259 (36.2)	120,727 (40.1)	152,247 (37.7)	36,036 (40.6)	28,065 (43.3)	10,017 (36.2)
High	669,115 (41.4)	341,737 (58.4)	25,858 (53.6)	143,472 (60.9)	172,407 (57.3)	240,368 (59.6)	50,480 (56.9)	34,514 (53.2)	16,375 (59.2)
Missing	122,767 (7.6)	859 (0.1)	166 (0.3)	251 (0.1)	442 (0.1)	333 (0.1)	171 (0.2)	187 (0.3)	168 (0.6)
Highest annual household income level–no. (%)^d^									
Low	195,872 (12.1)	46,959 (8.0)	4744 (9.8)	19,438 (8.3)	22,777 (7.6)	33,391 (8.3)	6470 (7.3)	5254 (8.1)	1841 (6.7)
Middle	598,182 (37.0)	211,963 (36.3)	18,751 (38.8)	88,081 (37.4)	105,131 (34.9)	149,259 (37.0)	30,722 (34.6)	23,293 (35.9)	8683 (31.4)
High	695,741 (43.1)	325,223 (55.6)	24,676 (51.1)	127,712 (54.3)	172,835 (57.4)	220,548 (54.7)	51,445 (58.0)	36,247 (55.9)	16,966 (61.3)
Missing	126,024 (7.8)	531 (0.1)	101 (0.2)	165 (0.1)	265 (0.1)	227 (0.1)	69 (0.1)	67 (0.1)	168 (0.6)
Maternal history of high-grade cervical lesions–no. (%)^d^									
No	1,547,188 (95.8)	551,717 (94.4)	45,728 (94.7)	220,300 (93.6)	285,689 (94.9)	379,246 (94.0)	84,383 (95.1)	61,692 (95.1)	26,396 (95.4)
Yes	68,631 (4.2)	32,959 (5.6)	2544 (5.3)	15,096 (6.4)	15,319 (5.1)	24,205 (6.0)	4323 (4.9)	3169 (4.9)	1262 (4.6)

IQR, interquartile range.

a Received at least one dose of quadrivalent HPV vaccination.

b The median age at first vaccination (IQR): one-dose, 14.1 (12.0–17.6); two-dose, 11.6 (11.2–12.4); three-dose, 14.5 (12.3–16.7).

c The median months (IQR) between the 1st and 2nd doses is 6.4 (6.0–6.9).

d Percentages may not total 100 because of rounding.

### HPV vaccination and the risk of high-grade cervical lesions

Among girls and women who received at least one dose of quadrivalent HPV vaccine, the overall IRR for high-grade cervical lesions of comparing vaccinated participants with unvaccinated participants was 0.62 (95% CI 0.60–0.63) (Table 2). When stratified by age at vaccination, the fully adjusted IRRs for vaccinated at 10–14, 15–16, 17–20, and 21–35 years of age compared to unvaccinated were 0.50 (95% CI 0.47–0.52), 0.54 (95% CI 0.52–0.56), 0.66 (95% CI 0.64–0.68), and 0.89 (95% CI 0.85–0.94), respectively. When stratified by number of doses received, compared to unvaccinated women, the overall fully adjusted IRRs for one, two, and three doses were 0.73 (95% CI 0.69–0.78), 0.70 (95% CI 0.66–0.74), and 0.59 (95% CI 0.58–0.61), respectively.

**Table 2:** HPV vaccination and high-grade cervical lesions (HCL) by doses received or age at vaccination.^d^

HPV vaccination status	No. of cases of HCL	Person-years (py)	Crude incidence rate per 1000 py (95% CI)	Age-adjusted incidence rate ratio (95% CI)^b^	Adjusted incidence rate ratio (95% CI)^c^
Unvaccinated	71,478	16,474,712	4.34 (4.31–4.37)	Reference	Reference
Vaccinated^a^	9909	4,845,737	2.04 (2.01–2.09)	0.77 (0.75–0.78)	0.62 (0.60–0.63)
Age at first vaccination, years					
10–14	1904	2,977,808	0.64 (0.61–0.67)	0.63 (0.61–0.66)	0.50 (0.47–0.52)
15–16	3039	971,030	3.13 (3.02–3.24)	0.69 (0.66–0.72)	0.54 (0.52–0.56)
17–20	3352	690,101	4.86 (4.70–5.02)	0.82 (0.79–0.85)	0.66 (0.64–0.68)
21–35	1614	206,797	7.80 (7.43–8.19)	1.07 (1.01–1.12)	0.89 (0.85–0.94)
Dosage					
One-dose	1049	564,759	1.86 (1.75–1.97)	0.90 (0.84–0.95)	0.73 (0.69–0.78)
Two-dose	1410	1,385,552	1.02 (0.97–1.07)	0.86 (0.82–0.91)	0.70 (0.66–0.74)
Three-dose	7450	2,895,426	2.57 (2.52–2.63)	0.74 (0.72–0.76)	0.59 (0.58–0.61)

a Received at least one dose of quadrivalent HPV vaccination.

b Adjusted for age as spline with 3 degrees of freedom.

c Adjusted for age as a spline term with 3 degrees of freedom, calendar year, county of residence, maternal history of high-grade cervical lesions, mother’s country of birth, highest parental education level, and highest annual household income level.

d With 1 year buffer period.

**Fig. 2:**
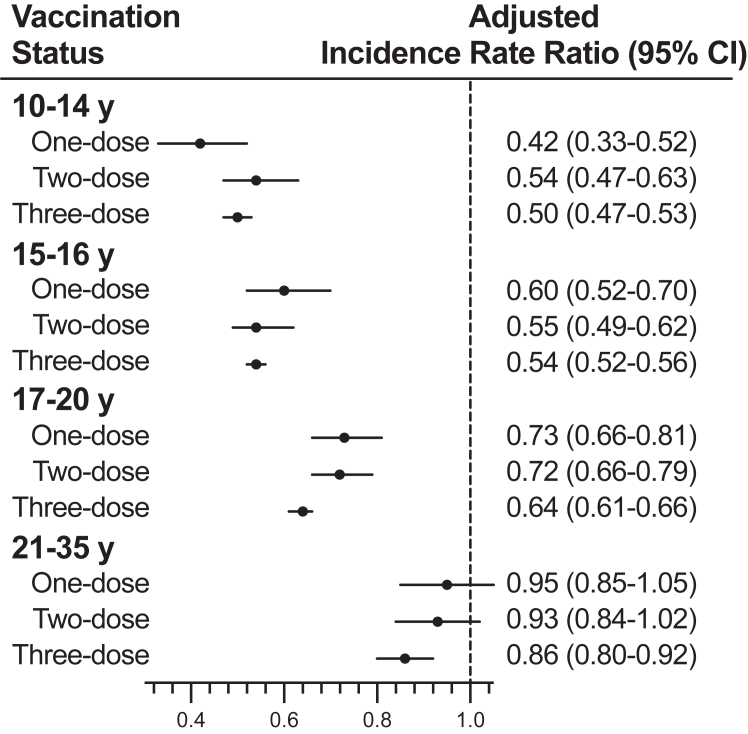
**HPV vaccination and risk ofhigh-gradecervical lesions by age at vaccination and doses received.** The adjusted incidence rate ratios with 95% confidence interval (CI) were adjusted for age as a spline term with 3 degrees of freedom, calendar year, county of residence, maternal history of high-grade cervical lesions, mother’s country of birth, highest parental education level, and highest annual household income level, with 1 year buffer, compared with unvaccinated group. The median interval (interquartile range) between the 1st and 2nd dose among girls or women who only received 2 doses was 6.4 (6.1–6.9) months for 10–14 y group, 3.3 (2.1–5.9) months for 15–16 y, 2.6 (2.0–5.5) months for 17–20 y and 2.4 (2.0–4.5) months for 21-35 y.

When examined by age at first vaccination and the number of doses received during our study period, we found a reduced risk of high-grade cervical lesions among women who initiated vaccination before age 17 regardless of doses received (Fig. 2 & Appendix p 6) compared with the unvaccinated group. We observed IRRs of 0.42 (95% CI 0.33–0.52), 0.54 (95% CI 0.47–0.63), and 0.50 (95% CI 0.47–0.53) among girls vaccinated with one, two, and three doses before age 15. While among those vaccinated at age 15–16 years, the reduced risk associated with one dose (IRR 0.60, 95% CI 0.52–0.70) was comparable with that associated with two doses (IRR 0.55, 95% CI 0.49–0.62) or three doses (IRR 0.54, 95% CI 0.52–0.56) as well. For women initially vaccinated between the age of 17 and 20 years, the IRRs of one dose (0.73, 95% CI 0.66–0.81) and two doses (0.72, 95% CI 0.66–0.79) were similar, while the IRR of three doses was 0.64 (95% CI 0.61–0.66). Among females vaccinated after age 20 years, only those who had three doses had a statistically significant lower incidence of high-grade cervical lesions with an IRR of 0.86 (95% CI 0.80–0.92).

### Sensitivity analysis

The overall, dose-specific, and age-specific effectiveness of the vaccine against high-grade cervical lesions showed a slight increase but remained comparable when buffer periods of 6, 12, 18, or 24 months were applied, compared to when no buffer period was used (Appendix p 7). Using various lengths of buffer period to examine the effectiveness by age and dose, we observed a significant reduced risk of high-grade cervical lesions among women vaccinated after age 20 and had only two doses when a buffer period of 18 and 24 months were used (Appendix p 8). In the complete-case analyses, the estimates are almost identical to the main results (Appendix p 9). In the subgroup analyses by excluding varying birth cohorts, the fully adjusted IRRs remained comparable with the main results (Appendix p 10), demonstrating that our main findings are robust to variations in vaccine coverage and screening attendance. Using different ages as cut-off points, the trend of more pronounced risk reduction when vaccinating at a younger age remained (Appendix p 11).

## Discussion

In this Swedish population-based cohort study, we observed that girls who received either one dose or two doses of the vaccines prior to age 17, especially before age 15, had comparable effectiveness against high-grade cervical lesions to girls who had received three doses. For women who initiated vaccination after age 20, the risk reduction was only statistically significant for women who received three doses with a 12-month buffer period. However, we found comparable effectiveness between women who had two and three doses in this group when a buffer period of 18 or 24 months was used (Appendix p 8). A lower incidence of high-grade cervical lesions was demonstrated in vaccinated women regardless of the dose of vaccination received or age at first vaccination compared with the unvaccinated girls and women. We observed a tendency of lower incidence among girls and women who were administered more doses or at a younger age.

The greater effectiveness after HPV vaccination at younger ages compared to older ages, as has been observed in many effectiveness studies,^4^^,^^12^^,^^23^ is likely due to a lower likelihood of prior natural HPV exposure from sexual activity before vaccination at younger ages.^12^ While lower antibody levels have been observed after vaccination at older ages, the minimum level of HPV antibodies that confers protection has not been established.^29^ Our observation that effectiveness varied by the number of doses in the older age groups could be largely due to differences in HPV exposure and sexual activity before vaccination. As we noted that, by applying a longer buffer period, we found comparable effectiveness between two and three doses for those vaccinated after age 20. Additionally, the effectiveness of two doses for those who were vaccinated at older age was likely to be underestimated because many of those girls and women vaccinated under the three-dose regimen with the 1st and 2nd doses less than 6 months apart.

Our findings were aligned with earlier observational studies and clinical trials. A systematic review by Ellingson et al. included 21 studies analyzing HPV vaccine effectiveness by age at vaccination and concluded that vaccine effectiveness against HPV-related diseases decreases with increasing age at initiation or completion of vaccination.^12^ A recent meta-analysis included 1,043,346 participants from 4 studies, all of which found comparable effectiveness against cervical intraepithelial neoplasia grades 2/3 by number of doses.^13^ However, there was a high heterogeneity in these studies, and the study population consisted mainly of girls younger than 16 years of age with a follow-up period of up to 8 years.^13^ Furthermore, most of those studies have limited precision when stratifying by age and doses.^13^ Recent randomized controlled trials focusing on single-dose HPV vaccination demonstrated sufficient immune response^9^^,^^10^ and efficacy^8^ of a one-dose vaccine in women aged 9–20 years to prevent HPV infection. Our results further extend the knowledge of the effectiveness of quadrivalent HPV vaccine against high-grade cervical lesions based on refined age at vaccination initiation according to the WHO recommendation. These results are also sufficiently powered to explore the dose-specific effectiveness through a 17-year follow-up after the introduction of HPV vaccination programs.

Our study has several strengths. In this large-scale population-based study, we have sufficient statistical power to reliably estimate the age- and dose-specific effectiveness through a real-life vaccination program. The extensive follow-up period of up to 17 years enables the evaluation of long-term vaccine effectiveness. We also used high-quality Swedish nationwide registries, which allow for valid and reliable measurement of both HPV vaccine status and high-grade cervical lesions. We obtained data concerning a rich set of potential confounders, including attained age, calendar year, county of residence, and parental characteristics such as education, income, mother’s country of birth, and maternal history of high-grade cervical lesions. The adjustment for these factors in our model allowed for the minimization of their confounding effects. We then performed several sensitivity analyses to ensure our estimates were robust. This included the use of various buffer periods to rule out prevalent infection, which may otherwise overestimate the incidence, especially among one- or two-dose groups.^12^^,^^24^ Moreover, our study also included analyses of varying subgroups to examine the potential bias introduced by lower vaccination or screening coverages and further test the robustness of our findings.

Nonetheless, there are also some limitations that are important to consider. *First*, a small proportion of vaccinated women (8% of all doses between 2006 and 2015) have been misclassified as unvaccinated due to anonymous records from SVEVAC (lack of informed consent).^4^ However, such misclassification will bias our estimates toward the null. *Second*, the vaccination records from an ongoing concomitant vaccination and screening campaign at age 23, which targeted birth cohorts 1994–1999 from 2021 onward, were not recorded in our study.^30^ However, lacking those vaccination records will mainly affect our estimates for women who were vaccinated after age 20. Given that the campaign began in 2021, initially limited to the Stockholm region, and our study follow-up concluded in 2022, the campaign’s impact on our findings is likely to be minimal. *Third*, vaccinated women might be more engaged in screening, leading to higher cervical lesion detection in these groups.^31^ This is addressed in our sensitivity analyses with selected birth cohorts, where screening attendance showed limited impact on vaccine effectiveness estimates after control for all covariates. *Fourth*, we were not able to further divide the outcomes by severity, i.e., cervical intraepithelial neoplasia grade 3 or worse, due to the classification changes for histopathological diagnoses in Sweden since 2017. *Fifth*, this is an observational study, women who received HPV vaccines may generally be healthier compared to those who did not (“healthy volunteer bias”). To address this, we adjusted for a comprehensive set of covariates that could influence both vaccine uptake and the underlying risk of high-grade cervical lesions. However, it is important to acknowledge that we cannot fully exclude the possibility of residual confounding from not being able to control for lifestyle factors, such as smoking and sexual activity, because the information is not available in registers. We consider our adjustment for parental education and household income levels could serve as partial proxies for these lifestyle variables.^4^^,^^27^ Information on ethnicity is also lacking from Swedish registers, and as an alternative, we have controlled for mother’s country of birth as a rough proxy. *Sixth*, though we have excluded recent immigrants, we assessed its impact for the validity of our study is limited. As it is known that Sweden has a universal healthcare system with comparatively equitable healthcare access.^32^ Evidence suggested that women born outside of Sweden did not have an excess risk of cervical diseases compared to those born in Sweden, nor significant differences in screening participation, after adjusting for education and income.^33^^,^^34^ Of note, we have included all eligible individuals who immigrated to Sweden before 2006, and 15% of our study population have a mother born outside Sweden. *Last*, although this is a large-scale study, statistical power remained limited to investigate further the timing between the 1st and 2nd doses after stratifying by age and dose. The risk reduction of having two doses of HPV vaccine might be underestimated, because many women had their 1st and 2nd doses less than 6 months apart, especially among women who initiated at an older age.

Our findings strongly support the importance of vaccination at an early age against HPV. Also, the findings support routine HPV immunization programs for teenagers globally to eliminate cervical cancer and further demonstrate the protection of reduced-dose (two- or one-dose) in adolescents at the population level. As countries switch from a two-dose to a one-dose schedule for routine vaccination programs in early ages,^3^ our findings provide evidence of comparable effectiveness against high-grade cervical lesions after one dose administered before age 15, which is in line with the WHO strategy. Our results are likely generalizable to countries with established vaccination and cervical screening programs that have similar coverage such as in many high-income countries. Since our study is population-based and includes women from diverse demographic and socioeconomic backgrounds, the findings should be relevant to populations with varying socioeconomic and cultural contexts. Reduced-dose regimens, especially for low- and middle-income countries, would increase vaccine access and facilitate scale-up of vaccination programs.^35^^,^^36^ Ultimately, increasing uptake among girls by 15 years old will help to achieve the WHO’s cervical cancer elimination goal.^35^^,^^36^ We did observe greater (if limited) protection among women vaccinated after age 17, especially after age 21, with higher doses. It emphasizes the importance of monitoring the vaccine effectiveness of a reduced-dose regimen, and continuously evaluating if and when a booster dose might be needed through investigating the vaccine effectiveness by length of follow-up time. Moreover, future studies could further explore the effectiveness of the other vaccine types used and the impact of the interval between doses.

In conclusion, the quadrivalent HPV vaccine has continued to demonstrate remarkable effectiveness in preventing high-grade cervical lesions up to 17 years after the introduction of HPV vaccination. Receiving one or two doses of HPV vaccines prior to age 17, especially for those initiating before age 15, has demonstrated comparable effectiveness with those who received 3 doses, which supports WHO’s current recommendation of using a one-dose HPV vaccine regimen in adolescents.

## Contributors

JL had conceptualized and designed the study, and contributed to funding acquisition, methodology, resources, supervision. SW did the formal analysis, methodology, visualization, and writing-original draft. JL and SW accessed and verified the underlying study data. AP contributed to data interpretation and methodology. AMAA, YD, and LAS interpreted the data. All authors critically reviewed and edited the manuscript and were jointly responsible for the decision to submit the manuscript for publication.

## Data sharing statement

The raw datasets are not available for sharing because of privacy policies and regulations in Sweden. Additional data is available on request from the corresponding author for any interested researchers provided all ethical and legal requirements are met.

## Declaration of interests

We declare no competing interests.
