# Supplementary material for: Effectiveness of quadrivalent human papillomavirus vaccination against high-grade cervical lesions by age and doses: a population-based cohort study
Source: Lancet Reg Health Eur. 2025 Jan 5;49:101178. doi: 10.1016/j.lanepe.2024.101178 (PMC11846428; doi:10.1016/j.lanepe.2024.101178)
Supplement: Appendix Figs. S1 and S2 and Tables S1–S8 [file mmc1.pdf]

Supplementary Appendix

Figure S1: Illustration of time-varying exposure and allocation of person-time ........................... 2

Figure S2: Illustration of buffer period, allocation of person-time ................................................ 3

Table S1: Summary of missing-values patterns ............................................................................. 4

Table S2: Characteristics of different birth cohorts ....................................................................... 5

Table S3: HPV vaccination and risk of high-grade cervical lesions (HCL) by age at vaccination and doses received * .................................................................................................................. 6

Table S4: HPV vaccination and risk of high-grade cervical lesions (HCL) by doses and by age at first vaccination, including buffer period of 6 to 24 months ......................................................... 7

Table S5: Incidence rate ratio of high-grade cervical lesions by age at vaccination and doses received compared with unvaccinated group, including buffer period of 6 to 24 months ............. 8

Table S6: Complete-case analysis of HPV vaccination and risk of high-grade cervical lesions (HCL) by age at vaccination and doses received * ........................................................................ 9

Table S7: HPV vaccination and risk of high-grade cervical lesions (HCL) among selected birth cohorts * ......................................................................................................................................... 10

Table S8: The age at HPV vaccination and risk of high-grade cervical lesions (HCL) by age at first vaccination * ........................................................................................................................... 11

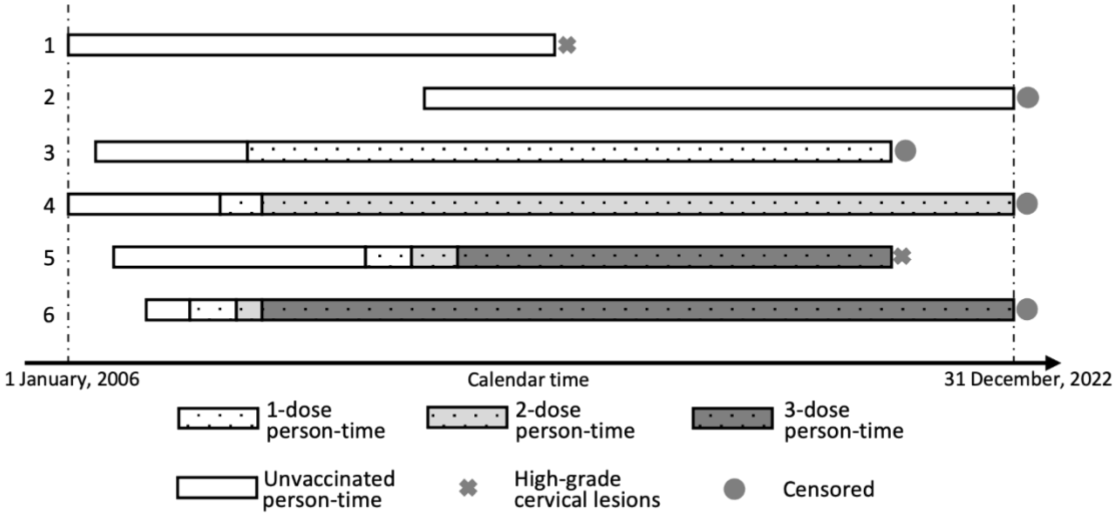


**Figure S1: Illustration of time-varying exposure and allocation of person-time**

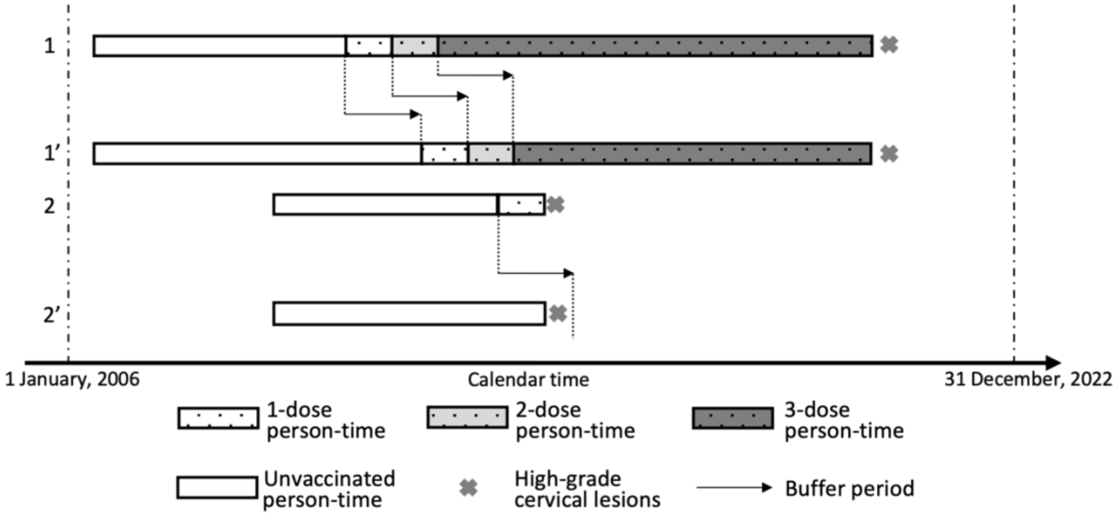


**Figure S2: Illustration of buffer period, allocation of person-time**

**Table S1: Summary of missing-values patterns**

| Number of Missing Variables [a] | Percentage of individuals [b] |
|---|---|
| 0 | 93·5% |
| 1 | 1·1% |
| 2 | 0·1% |
| 3 | 3·4% |
| 4 | 2·0% |

a. Variables include county of residence, highest parental education level, highest annual household income level, and mother's country of birth
b. Percentages may not total 100 because of rounding.

Among the study population, 93·5% of individuals have complete information for covariates including county of residence, highest parental education level, highest annual household income level, and mother's country of birth.

**Table S2: Characteristics of different birth cohorts**

| Birth Cohort | Vaccination Status | No. of Cases of HCL | Person-years (py) | qHPV Vaccination coverage, % | Median Vaccination age (IQR) | Median follow-up years (IQR) |
|---|---|---|---|---|---|---|
| 1970-1979 | Unvaccinated | 10,972 | 2,483,076 | 0·13 | 33 (31,35) | 4 (2,7) |
| | Vaccinated [a] | 19 | 2,222 | | | |
| 1980-1989 | Unvaccinated | 42,936 | 6,697,574 | 4·73 | 25 (21,26) | 14 (12,17) |
| | Vaccinated [a] | 1,670 | 247,985 | | | |
| 1990-1994 | Unvaccinated | 14,458 | 3,272,376 | 40·67 | 17 (16,18) | 17 (17,17) |
| | Vaccinated [a] | 5,799 | 1,483,410 | | | |
| 1995-1999 | Unvaccinated | 2,724 | 2,035,264 | 59·38 | 14 (13,16) | 16 (14,17) |
| | Vaccinated [a] | 2,728 | 1,464,630 | | | |
| 2000-2004 | Unvaccinated | 24 | 735,133 | 82·82 | 12 (11,12) | 10 (9,12) |
| | Vaccinated [a] | 75 | 1,646,663 | | | |
| 2005-2012 | Unvaccinated | 0 | 665,849 | 28·40 | 11 (11,12) | 2 (1,6) |
| | Vaccinated [a] | 2 | 586,269 | | | |
| Total | Unvaccinated | 71,114 | 15,889,271 | 26·57 | 13 (11,16) | 10 (4,16) |
| | Vaccinated [a] | 10,273 | 5,431,178 | | | |

IQR, interquartile range

a. Received at least one dose of quadrivalent HPV vaccination.

**Table S3: HPV vaccination and risk of high-grade cervical lesions (HCL) by age at vaccination and doses received ***

| HPV Vaccination Status | No. of Cases of HCL | Person-years (py) | Crude Incidence Rate per 1,000 py (95% CI) | Age-adjusted Incidence Rate Ratio (95% CI) [a] | Adjusted Incidence Rate Ratio (95% CI) [b] |
|---|---|---|---|---|---|
| **Unvaccinated** | 71,478 | 16474712 | 4·34 (4·31-4·37) | Reference | Reference |
| **Age at first vaccination, years** | | | | | |
| **10-14 y** | | | | | |
| **One-dose** | 80 | 350,251 | 0·23 (0·18-0·28) | 0·52 (0·42-0·65) | 0·42 (0·33-0·52) |
| **Two-dose** | 182 | 1,094,255 | 0·17 (0·14-0·19) | 0·68 (0·59-0·79) | 0·54 (0·47-0·63) |
| **Three-dose** | 1,642 | 1,533,302 | 1·07 (1·02-1·12) | 0·64 (0·61-0·67) | 0·50 (0·47-0·53) |
| **15-16 y** | | | | | |
| **One-dose** | 189 | 82,196 | 2·30 (1·99-2·65) | 0·77 (0·67-0·89) | 0·60 (0·52-0·70) |
| **Two-dose** | 301 | 128,852 | 2·34 (2·09-2·62) | 0·70 (0·62-0·78) | 0·55 (0·49-0·62) |
| **Three-dose** | 2,549 | 759,982 | 3·35 (3·23-3·49) | 0·68 (0·66-0·71) | 0·54 (0·52-0·56) |
| **17-20 y** | | | | | |
| **One-dose** | 405 | 87,406 | 4·63 (4·20-5·11) | 0·91 (0·82-1·00) | 0·73 (0·66-0·81) |
| **Two-dose** | 527 | 114,005 | 4·62 (4·24-5·03) | 0·89 (0·82-0·98) | 0·72 (0·66-0·79) |
| **Three-dose** | 2,420 | 488,691 | 4·95 (4·76-5·15) | 0·80 (0·76-0·83) | 0·64 (0·61-0·66) |
| **21-35 y** | | | | | |
| **One-dose** | 375 | 44,906 | 8·35 (7·55-9·24) | 1·12 (1·02-1·24) | 0·95 (0·85-1·05) |
| **Two-dose** | 400 | 48,440 | 8·26 (7·49-9·11) | 1·11 (1·01-1·22) | 0·93 (0·84-1·02) |
| **Three-dose** | 839 | 113,452 | 7·40 (6·91-7·91) | 1·02 (0·96-1·10) | 0·86 (0·80-0·92) |

* With 1 year buffer period.

a. Adjusted for age as spline with 3 degrees of freedom.

b. Adjusted for age as a spline term with 3 degrees of freedom, calendar year, county of residence, maternal history of high-grade cervical lesions, mother's country of birth, highest parental education level, and highest annual household income level.

**Table S4: HPV vaccination and risk of high-grade cervical lesions (HCL) by doses and by age at first vaccination, including buffer period of 6 to 24 months**

| Buffer Period | HPV Vaccination Status | No. of Cases of HCL | Age-adjusted Incidence Rate Ratio (95% CI) [b] | Adjusted Incidence Rate Ratio (95% CI) [c] |
|---|---|---|---|---|
| | Unvaccinated | 71,114 | Ref. | Ref. |
| | Vaccinated [a] | 10,273 | 0·79 (0·77-0·80) | 0·64 (0·62-0·65) |
| | One-dose | 1,168 | 0·97 (0·91-1·03) | 0·80 (0·75-0·85) |
| | Two-dose | 1,472 | 0·88 (0·83-0·93) | 0·72 (0·68-0·76) |
| **No Buffer** | Three-dose | 7,633 | 0·75 (0·73-0·77) | 0·60 (0·59-0·62) |
| | 10-14 | 1,904 | 0·64 (0·61-0·67) | 0·50 (0·48-0·53) |
| | 15-16 | 3,041 | 0·69 (0·67-0·72) | 0·55 (0·52-0·57) |
| | 17-20 | 3,379 | 0·83 (0·80-0·86) | 0·67 (0·64-0·69) |
| | 21-35 | 1,949 | 1·13 (1·08-1·18) | 0·96 (0·91-1·00) |
| | Unvaccinated | 71,329 | Ref. | Ref. |
| | Vaccinated [a] | 10,058 | 0·77 (0·76-0·79) | 0·62 (0·61-0·64) |
| | One-dose | 1,079 | 0·91 (0·85-0·96) | 0·74 (0·70-0·79) |
| | Two-dose | 1,440 | 0·87 (0·82-0·91) | 0·71 (0·67-0·75) |
| **6 months** | Three-dose | 7,539 | 0·74 (0·72-0·76) | 0·60 (0·58-0·61) |
| | 10-14 | 1,904 | 0·64 (0·61-0·67) | 0·50 (0·47-0·52) |
| | 15-16 | 3,041 | 0·69 (0·67-0·72) | 0·54 (0·52-0·56) |
| | 17-20 | 3,368 | 0·83 (0·80-0·86) | 0·66 (0·64-0·69) |
| | 21-35 | 1,745 | 1·08 (1·03-1·13) | 0·91 (0·86-0·95) |
| | Unvaccinated | 71,478 | Ref. | Ref. |
| | Vaccinated [a] | 9,909 | 0·77 (0·75-0·78) | 0·62 (0·60-0·63) |
| | One-dose | 1,049 | 0·90 (0·84-0·95) | 0·73 (0·69-0·78) |
| | Two-dose | 1,410 | 0·86 (0·82-0·91) | 0·70 (0·66-0·74) |
| **12 months** | Three-dose | 7,450 | 0·74 (0·72-0·76) | 0·59 (0·58-0·61) |
| | 10-14 | 1,904 | 0·63 (0·61–0·66) | 0·50 (0·47–0·52) |
| | 15-16 | 3,039 | 0·69 (0·66–0·72) | 0·54 (0·52–0·56) |
| | 17-20 | 3,352 | 0·82 (0·79–0·85) | 0·66 (0·64–0·68) |
| | 21-35 | 1,614 | 1·07 (1·01–1·12) | 0·89 (0·85–0·94) |
| | Unvaccinated | 71,611 | Ref. | Ref. |
| | Vaccinated [a] | 9,776 | 0·76 (0·75-0·78) | 0·61 (0·60-0·63) |
| | One-dose | 1,049 | 0·91 (0·86-0·97) | 0·74 (0·70-0·79) |
| | Two-dose | 1,357 | 0·84 (0·80-0·89) | 0·68 (0·65-0·72) |
| **18 months** | Three-dose | 7,370 | 0·74 (0·72-0·75) | 0·59 (0·57-0·60) |
| | 10-14 | 1,904 | 0·63 (0·61-0·66) | 0·50 (0·47-0·52) |
| | 15-16 | 3,037 | 0·69 (0·66-0·72) | 0·54 (0·52-0·56) |
| | 17-20 | 3,339 | 0·82 (0·79-0·85) | 0·66 (0·63-0·68) |
| | 21-35 | 1,496 | 1·07 (1·01-1·12) | 0·89 (0·84-0·94) |
| | Unvaccinated | 71,778 | Ref. | Ref. |
| | Vaccinated [a] | 9,609 | 0·76 (0·74-0·77) | 0·61 (0·59-0·62) |
| | One-dose | 1,007 | 0·89 (0·84-0·95) | 0·73 (0·68-0·77) |
| | Two-dose | 1,315 | 0·82 (0·78-0·87) | 0·67 (0·63-0·71) |
| **24 months** | Three-dose | 7,287 | 0·73 (0·71-0·75) | 0·59 (0·57-0·60) |
| | 10-14 | 1,904 | 0·63 (0·61-0·66) | 0·50 (0·47-0·52) |
| | 15-16 | 3,035 | 0·69 (0·66-0·71) | 0·54 (0·52-0·56) |
| | 17-20 | 3,313 | 0·82 (0·79-0·85) | 0·65 (0·63-0·68) |
| | 21-35 | 1,357 | 1·05 (1·00-1·11) | 0·87 (0·83-0·92) |

a. Received at least one dose of quadrivalent HPV vaccination.
b. Adjusted for age as spline with 3 degrees of freedom.
c. Adjusted for age as a spline term with 3 degrees of freedom, calendar year, county of residence, maternal history of high-grade cervical lesions, mother's country of birth, highest parental education level, and highest annual household income level.

**Table S5: Incidence rate ratio of high-grade cervical lesions by age at vaccination and doses received compared with unvaccinated group, including buffer period of 6 to 24 months**

| HPV Vaccination Status | 0 month | 6 months | 12 months | 18 months | 24 months |
|---|---|---|---|---|---|
| **10-14 y** | | | | | |
| **One-dose** | 0·42 (0·33-0·52) | 0·42 (0·33-0·52) | 0·42 (0·33-0·52) | 0·42 (0·33-0·52) | 0·42 (0·33-0·52) |
| **Two-dose** | 0·54 (0·47-0·63) | 0·54 (0·47-0·63) | 0·54 (0·47-0·63) | 0·54 (0·47-0·63) | 0·54 (0·47-0·62) |
| **Three-dose** | 0·50 (0·48-0·53) | 0·50 (0·47-0·53) | 0·50 (0·47-0·53) | 0·50 (0·47-0·53) | 0·50 (0·47-0·53) |
| **15-16 y** | | | | | |
| **One-dose** | 0·61 (0·52-0·70) | 0·61 (0·53-0·70) | 0·60 (0·52-0·70) | 0·60 (0·52-0·70) | 0·60 (0·52-0·69) |
| **Two-dose** | 0·55 (0·49-0·62) | 0·55 (0·49-0·61) | 0·55 (0·49-0·62) | 0·55 (0·49-0·61) | 0·54 (0·49-0·61) |
| **Three-dose** | 0·54 (0·52-0·56) | 0·54 (0·52-0·56) | 0·54 (0·52-0·56) | 0·54 (0·52-0·56) | 0·54 (0·52-0·56) |
| **17-20 y** | | | | | |
| **One-dose** | 0·74 (0·67-0·82) | 0·73 (0·66-0·81) | 0·73 (0·66-0·81) | 0·74 (0·67-0·81) | 0·71 (0·64-0·78) |
| **Two-dose** | 0·73 (0·67-0·79) | 0·73 (0·67-0·80) | 0·72 (0·66-0·79) | 0·72 (0·66-0·78) | 0·71 (0·65-0·77) |
| **Three-dose** | 0·64 (0·62-0·67) | 0·64 (0·61-0·67) | 0·64 (0·61-0·66) | 0·63 (0·61-0·66) | 0·63 (0·61-0·66) |
| **21-35 y** | | | | | |
| **One-dose** | 1·12 (1·02-1·22) | 0·96 (0·87-1·06) | 0·95 (0·85-1·05) | 1·00 (0·90-1·11) | 0·99 (0·89-1·10) |
| **Two-dose** | 0·96 (0·87-1·05) | 0·93 (0·85-1·02) | 0·93 (0·84-1·02) | 0·87 (0·78-0·97) | 0·83 (0·74-0·93) |
| **Three-dose** | 0·89 (0·84-0·95) | 0·87 (0·82-0·93) | 0·86 (0·80-0·92) | 0·86 (0·80-0·92) | 0·84 (0·78-0·91) |

Incidence rate ratios were adjusted for age as a spline term with 3 degrees of freedom, calendar year, county of residence, maternal history of high-grade cervical lesions, mother's country of birth, highest parental education level, and highest annual household income level. Using the unvaccinated group as the reference.

**Table S6: Complete-case analysis of HPV vaccination and risk of high-grade cervical lesions (HCL) by age at vaccination and doses received ***

| HPV Vaccination Status | No. of Cases of HCL | Person-years (py) | Crude Incidence Rate per 1,000 py (95% CI) | Age-adjusted Incidence Rate Ratio (95% CI) [a] | Adjusted Incidence Rate Ratio (95% CI) [b] |
|---|---|---|---|---|---|
| **Unvaccinated** | 69,828 | 15,527,635 | 4·50 (4·46-4·53) | Reference | Reference |
| **Age at first vaccination, years** | | | | | |
| **10-14 y** | | | | | |
| **One-dose** | 80 | 348,938 | 0·23 (0·18-0·29) | 0·51 (0·41-0·63) | 0·42 (0·33-0·52) |
| **Two-dose** | 180 | 1,091,374 | 0·16 (0·14-0·19) | 0·65 (0·56-0·75) | 0·53 (0·46-0·62) |
| **Three-dose** | 1,635 | 1,527,712 | 1·07 (1·02-1·12) | 0·61 (0·58-0·64) | 0·50 (0·47-0·52) |
| **15-16 y** | | | | | |
| **One-dose** | 189 | 81,591 | 2·32 (2·01-2·67) | 0·74 (0·65-0·86) | 0·60 (0·52-0·70) |
| **Two-dose** | 300 | 127,990 | 2·34 (2·09-2·62) | 0·67 (0·60-0·75) | 0·55 (0·49-0·62) |
| **Three-dose** | 2,534 | 756,118 | 3·35 (3·22-3·48) | 0·65 (0·63-0·68) | 0·54 (0·51-0·56) |
| **17-20 y** | | | | | |
| **One-dose** | 402 | 86,476 | 4·65 (4·22-5·13) | 0·87 (0·79-0·96) | 0·73 (0·66-0·80) |
| **Two-dose** | 523 | 113,123 | 4·62 (4·24-5·04) | 0·86 (0·79-0·93) | 0·72 (0·66-0·78) |
| **Three-dose** | 2,414 | 485,762 | 4·97 (4·78-5·17) | 0·76 (0·73-0·80) | 0·64 (0·61-0·66) |
| **21-35 y** | | | | | |
| **One-dose** | 370 | 44,467 | 8·32 (7·51-9·21) | 1·06 (0·96-1·17) | 0·94 (0·85-1·04) |
| **Two-dose** | 396 | 47,900 | 8·27 (7·49-9·12) | 1·05 (0·95-1·16) | 0·92 (0·84-1·02) |
| **Three-dose** | 832 | 112,507 | 7·40 (6·91-7·92) | 0·97 (0·90-1·04) | 0·86 (0·80-0·92) |

* With 1 year buffer period.

a. Adjusted for age as spline with 3 degrees of freedom.

b. Adjusted for age as a spline term with 3 degrees of freedom, calendar year, county of residence, maternal history of high-grade cervical lesions, mother's country of birth, highest parental education level, and highest annual household income level.

**Table S7: HPV vaccination and risk of high-grade cervical lesions (HCL) among selected birth cohorts ***

| | HPV Vaccination Status | No. of Cases of HCL | Person-year (py) | Crude Incidence Rate per 1,000 py (95% CI) | Age-Adjusted Incidence Rate Ratio (95% CI) [e] | Adjusted Incidence Rate Ratio (95% CI) [f] |
|---|---|---|---|---|---|---|
| **1990-2012 [a]** | Unvaccinated | 17,341 | 7,269,585 | 2·39 (2·35-2·42) | Ref | Ref· |
| | Vaccinated [d] | 8,449 | 4,620,007 | 1·83 (1·79-1·87) | 0·61 (0·60-0·63) | 0·62 (0·60-0·63) |
| **1970-1999 [b]** | Unvaccinated | 71,454 | 14,767,036 | 4·84 (4·80-4·87) | Ref | Ref· |
| | Vaccinated [d] | 9,832 | 2,919,500 | 3·37 (3·30-3·43) | 0·77 (0·76-0·79) | 0·62 (0·60-0·63) |
| **1990-1999 [c]** | Unvaccinated | 17,317 | 5,561,910 | 3·11 (3·07-3·16) | Ref | Ref· |
| | Vaccinated [d] | 8,372 | 2,693,770 | 3·11 (3·04-3·18) | 0·62 (0·60-0·64) | 0·62 (0·60-0·64) |

* With 1 year buffer period.
a. 98.6% of individuals have complete information for all variables.
b. 91.0% of individuals have complete information for all variables.
c. 97.6% of individuals have complete information for all variables.
d. Received at least one dose of quadrivalent HPV vaccination.
e. Adjusted for age as spline with 3 degrees of freedom.
f. Adjusted for age as a spline term with 3 degrees of freedom, calendar year, county of residence, maternal history of high-grade cervical lesions, mother's country of birth, highest parental education level, and highest annual household income level.

**Table S8: The age at HPV vaccination and risk of high-grade cervical lesions (HCL) by age at first vaccination ***

| HPV Vaccination Status | No. of Cases of HCL | Person-years (py) | Crude Incidence Rate per 1,000 py (95% CI) | Age-adjusted Incidence Rate Ratio (95% CI) [a] | Adjusted Incidence Rate Ratio (95% CI) [b] |
|---|---|---|---|---|---|
| **Unvaccinated** | 71,478 | 16,474,712 | 4·34 (4·31-4·37) | Reference | Reference |
| **Age at first vaccination, years** | | | | | |
| **10-12** | 149 | 2,062,956 | 0·07 (0·06-0·08) | 0·33 (0·28-0·39) | 0·26 (0·22-0·31) |
| **13-14** | 1,755 | 914,852 | 1·92 (1·83-2·01) | 0·68 (0·65-0·72) | 0·54 (0·51-0·56) |
| **15-16** | 3,039 | 971,030 | 3·13 (3·02-3·24) | 0·69 (0·66-0·72) | 0·54 (0·52-0·56) |
| **17-20** | 3,352 | 690,101 | 4·86 (4·70-5·02) | 0·82 (0·79-0·85) | 0·66 (0·63-0·68) |
| **21-35** | 1,614 | 206,797 | 7·80 (7·43-8·19) | 1·07 (1·02-1·12) | 0·89 (0·85-0·94) |
| **Age at first vaccination, years** | | | | | |
| **10-16** | 4,943 | 3,948,838 | 1·25 (1·22-1·29) | 0·67 (0·65-0·69) | 0·53 (0·51-0·55) |
| **17-35** | 4,966 | 896,899 | 5·54 (5·38-5·69) | 0·89 (0·86-0·92) | 0·72 (0·70-0·74) |
| **Age at first vaccination, years** | | | | | |
| **10-14** | 1,904 | 2,977,808 | 0·64 (0·61-0·67) | 0·64 (0·61-0·67) | 0·50 (0·48-0·53) |
| **15-20** | 6,391 | 1,661,131 | 3·85 (3·75-3·94) | 0·75 (0·73-0·77) | 0·60 (0·58-0·62) |
| **21-35** | 1,614 | 2067,97 | 7·80 (7·43-8·19) | 1·07 (1·01-1·12) | 0·89 (0·85-0·94) |

* With 1 year buffer period.

a. Adjusted for age as spline with 3 degrees of freedom.

b. Adjusted for age as a spline term with 3 degrees of freedom, calendar year, county of residence, maternal history of high-grade cervical lesions, mother's country of birth, highest parental education level, and highest annual household income level.
